# Numerical model of the spatio-temporal dynamics in a water strider group

**DOI:** 10.1038/s41598-021-96686-w

**Published:** 2021-09-10

**Authors:** Alexander Kovalev, Alexander E. Filippov, Stanislav N. Gorb

**Affiliations:** 1 Functional Morphology and Biomechanics, Zoological Institute, Kiel University, Am Botanischen Garten, 1-9, 24118 Kiel, Germany; 2 Donetsk Institute for Physics and Engineering, National Academy of Sciences of Ukraine, Donetsk, Ukraine

**Keywords:** Computational models, Animal behaviour, Entomology, Ecological modelling

## Abstract

The water strider group demonstrates a very complex dynamics consisting of competition for the food items, territoriality and aggression to the conspecific individuals, escaping from the predators, etc. The situation is even more complex due to the presence of different instars, which in most water strider species live in the same habitat and occupy the same niche. The presented swarm model of water striders demonstrates the realistic population dynamics. For the swarm formation in the model, attraction and repulsion forces were used. Animal motion in the model takes into account inertia and kinetic energy dissipation effects. The model includes three different rates related to the growth of individuals: food appearance rate, food assimilation rate, and stored energy loss rate. The results of our modeling show that the size distribution of individuals seems to be an adequate measure for population status, and it has a characteristic shape for different model parameter combinations. Distribution of the distances between nearest neighbors is other important measure of the population density and its dynamics. Parameters of the model can be tuned in such a way, that the shape of both distributions in a steady phase coincides with that shape observed in a natural population, which helps to understand the factors leading to particular momentary distribution of both parameters (size and distance) in the population. From this point of view, the model can predict how both distributions can further develop from certain state depending on particular combination of factors.

## Introduction

The finite group of water striders *Gerris* occupying water body demonstrates a very complex dynamics consisting of competition for the food items, territoriality and aggression to the conspecific individuals, escaping from the predators, etc.^1^. The situation is even more complex due to the presence of different instars, which in most *Gerris* species live in the same habitat and occupy the same niche. However, in *Gerris argentatus*, the older stages have been found on more open water surface than the younger ones, and totally open water was avoided^2^.

Life cycle of water striders includes an egg stage, five larval instars, and the imago, which overwinters. Both adults and larvae feed on invertebrates (insects) on the water surface and that is why compete for food in the case of species, where different instars tend to share the same niche within the habitat. The success of the several generations of eggs that can be produced in a year depends on the presence of older individuals. For example, in natural populations of *Gerris najas* none of the later broods survived, but when the numbers of older striders were artificially reduced the survivorship of young nymphs improved^3^. It means that the momentary set of interactions between different instars of different size within the habitat leads to the long term effects and influences population dynamics within the habitat. Different authors tried to track experimentally the exact changes in the population structure of various *Gerris* species, but the link between such behavioral features as territoriality, competition for the food items and escaping from predators is difficult to establish in an experimental study. Therefore, we undertaken a numerical modeling approach, based on the data about the size distribution within a population and on the information about spatial distribution of individuals, in order to understand the role of various types of interactions between water strider individuals on the long-term population dynamics of different instars.

It is well known that the life cycles of water striders are rather complex due to the presence of dimorphism in wing length. The adaptive strategies of the life cycles were previously discussed in the literature^1^. In the species occupying the most permanent habitats almost complete aplerism has evolved. Species of permanent water demonstrate a genetically switched alary dimorphism. In the present study, we do not consider the presence of dimorphism and behavioral differences between sexes. Based on behavioral observations and video recordings, we established a simple, but realistic model of the behavioral dynamics in the group of water striders consisting of individuals with different dimensions within the population in the natural habitat (for an example of original video sequence, see video_S1.avi).

The idea of such a model is rather pioneering for swarming group of insects in 2D space. In contrast to the previous model of the psocopteran group of same-aged animals, living in “cylindrical” space on the tree trunk^4^, the group of water striders consists of individuals of different size and different age. Consistently, this group is expected to behave strongly dependent on the size distribution of individuals within the group. From evolutionary ecology point of view, it is important to explore (1) costs-and-benefits of the individual size in different behavioral situations, (2) long-term dynamics within the group, and (3) the effects of resources on the group behavior. The main strength of the model is in its ability to provide some predictions: how distributions of sizes and distances between individuals can develop from certain state depending on particular combination of factors. Additionally, the model can also be potentially adapted to populations of water striders of different species.

## Data acquisition

Videos (eight sequences) were taken in mid-August 2018 at the population of *Gerris lacustris* at a small pond between Trakai and Klaipeda (Lithuania, Lat./Long. coordinates: 54.802612, 24.498949) (Fig. S2A, video_S1.mp4). The pond is surrounded by vegetation, directly on the shore, about 2–3 m broad. Areas of totally open water surface without vegetation were present in the center of the pond. Due to the fact that the pond is situated in a slight depression, the pond is sheltered from winds. During our video recordings (11:00–12:00 a.m.), a small-scaled patchwork of shade and sunshine was present at the study site. The videos were processed and analyzed carefully not to neglect even the smallest instars.

The processing and analysis of videos included following steps. The coordinate transformation was performed according to the following equations (Fig. S1):

1 $$\begin{aligned} y_{r} & = h\left[ {\cot \left( {\alpha + \tan^{ - 1} \left( {\frac{{2y_{c} - y_{cm} }}{{x_{cm} }}\tan \frac{\beta }{2}} \right)} \right) - \cot \alpha } \right], \\ x_{r} & = \frac{{2x_{c} - x_{cm} }}{{x_{cm} }}h\sqrt {1 + \left( {\frac{{y_{r} }}{h} + \cot \alpha } \right)^{2} } \tan \left( {\beta /2} \right), \\ \end{aligned}$$

where (*x*_r_, *y*_r_) are coordinates in the field, (*x*_c_, *y*_c_) are the camera coordinates, *x*_cm_ and *y*_cm_ are the horizontal and vertical camera resolution, *α* is an angle between ground plane and the normal to the camera plane, *β* is the view angle of camera in horizontal direction, *h* is the camera height over the ground. Surface water waves, their round shape, and their propagation were used to determine the unknown angle (*α*) and the height (*h*) for proper coordinates’ transformation. The surface water wave velocity is known to be 0.24 m/s. An image in field coordinates after coordinate transformation is presented in Fig. S2B.

The body of water striders were painted over in GIMP (ver. 2.10.8) (Fig. S2C) and analyzed using Matlab (MathWorks). The body volume was calculated as the volume a of a two-axis ellipsoid. The result is precise with some factor, which depends on the ratio between body thickness and body width.

The histograms of body weight and distances between nearest neighbors were calculated.

## Numerical model

The model is organized as follows. We simulated water striders as an array of the discrete “objects” which interact one with another according to more or less natural rules. The interaction includes strong short-range repulsion between the animals. Mathematically, short range repulsion means that one animal cannot penetrate inside a private area of other animal. Normally such repulsion appears at relatively short distances corresponding to a radius $R^{repuls}$ of their private territory. Besides, there is mutual attraction at longer distances $R^{attract} > R^{repuls}$, which biologically corresponds to a tendency to aggregation^4^. The tendency to aggregation often gives some competitive advantage due to possible collective reactions on the external challenges (for example, on the attacks of predators).

The simplest way to simulate an interaction with regulated characteristic distance is to use Gaussian effective potential with some characteristic radius $R_{0}$, since the Gaussian potential guaranty the stability in dynamics simulations with relatively large $\Delta t$^4^. Repulsion force in this case looks as follows:

2 $$f_{j}^{rep} = A_{0} \left( {\vec{r} - \vec{r}_{j} } \right)exp\left[ { - \left( {\frac{{\vec{r} - \vec{r}_{j} }}{{R_{0} }}} \right)^{2} } \right],$$

where factor *A*_0_ < 0 defines an amplitude of the attraction, vector $\vec{r} = \left\{ {x,y} \right\}$ describes a position of some particular animal and the array of the vectors $\overrightarrow {r}_{j} = \{ x_{j} ,y_{j} \}$ describe the positions of all other animals numerated by index $j = 1, \ldots N - 1$ with total number of them in the population *N*.

In the problem under consideration the animals generally have different size and strength, which can be modeled with the repulsion forces amplitude $B_{0j}$ and radius $R_{0j}$:

3 $$f_{{_{jk} }}^{repuls} (\overrightarrow {r}_{j} ,\overrightarrow {r}_{k} ) = B_{{_{jk} }}^{repuls} (\overrightarrow {r}_{j} - \overrightarrow {r}_{k} )\exp \left[ { - \left( {\frac{{\overrightarrow {r}_{j} - \overrightarrow {r}_{k} }}{{R_{{_{jk} }}^{repuls} }}} \right)^{2} } \right],$$

In general case the parameters of this interaction and their dispersion can vary from one population to another around some mean value typical for the particular species.

The tendency to aggregate is simulated by a mutual attraction at relatively long distances. Corresponding force can be written in the following form:

4 $$f_{{_{jk} }}^{attract} (\overrightarrow {r}_{j} ,\overrightarrow {r}_{k} ) = B_{{_{jk} }}^{attract} (\overrightarrow {r}_{j} - \overrightarrow {r}_{k} )\exp \left[ { - \left( {\frac{{\overrightarrow {r}_{j} - \overrightarrow {r}_{k} }}{{R_{{_{jk} }}^{attract} }}} \right)^{2} } \right],$$

where the coefficient $B_{{_{jk} }}^{attract}$ before the exponent regulates the attraction strength, and $R_{{_{jk} }}^{attract}$ characterizes the effective distance of the attraction.

The combination of the strong short-range repulsion and weak $B^{attract} < B^{repuls}$ but long-range $R^{attract} > R^{repuls}$ attraction normally causes a minimum of the interaction energy at some intermediate distance $R^{\min }$. In infinite empty space, being used in the equations of motion, such a combination of the forces leads to an ordering of the objects with the equilibrium distance corresponding to the position $R_{\min }$ of the energy minimum. But, if the area $\{ [0,Lx],[0,Ly]\}$ is limited, the equations of motion must be supplied by appropriate boundary conditions which do not allow the animals to leave this area.

The simplest way to introduce the boundary conditions is to apply mathematically “soft” but extremely high and narrow walls around the system, which repulse the animals back to the internal space with exponentially growing force

5 $$f_{j}^{bound} = B_{j}^{bound} \exp \left[ { - \left| {\frac{{\overrightarrow {r}_{j} - \overrightarrow {r}_{bound} }}{{R_{{}}^{bound} }}} \right|} \right]$$

acting in the direction opposite to that in which the animal occasionally crosses any of the boundaries. The words “extremely high” mean that the amplitude of this force should be supplied by the pre-factor $B_{j}^{bound}$ which is much bigger than the amplitude of the repulsion force between the animals (regulated by the amplitude $B_{{_{jk} }}^{repuls}$) to be able to overpower their mutual repulsion $B_{j}^{bound} > > B_{j}^{repuls}$ pushing them out the boundaries. Therefore also the characteristic distance of the repulsion from the wall should be much shorter than the typical distance between the animals in the population $R^{bond} < < R^{\min }$. In this sense the boundary should be as narrow as possible.

One can expect that mean density of the population is not extremely high and does not force the animals to stay exactly on the minimal distance $R^{\min }$ between them. In this case total combination of the attracting and repulsing interactions combined with the rejecting boundary conditions normally leads to the specific patterns where relatively dense groups of the animals spread on the distances close to the equilibrium ones are accompanied by almost empty voids between them^5^. We have checked this hypothesis numerically many times. Below, such patterns will be seen in all the particular visualizations of the numerical results.

It should be noted that mathematically, if number of the animals increases inside of the same limited and already populated area, the individuals tend to fill all the voids with the equilibrium (sometimes, even higher than equilibrium) density. It happens in real population as well in that cases when the number of the animals grows quickly for a self-regulation of the population and they become simply forced to occupy every empty space inside the area.

However, normally when the population grows too quickly the growth should be regulated by a number of factors. In particular, it will be restricted by a competition for the food and space accompanied by death of some of the participants of the process. From mathematical point of view it means that the equations of motion, which will be written below, must be accompanied by natural generation of the new individuals as well as by their (reasonable) disappearance from the system.

It is almost impossible to write such a generation in analytical form, but in numerical model it can be formally presented as a set of natural rules. First of all the length of the array is supposed to be a variable integer. Let us suppose that at every step of calculation $\Delta t$ the length of the array can potentially increase to the new one $N \to N + 1$. The potential act of the generation becomes real if the numerically generated random number $\varsigma$ uniformly distributed in interval [0;1] is less than some threshold $\varsigma_{thres} < 1$. In the numerical procedure the rate of the generation is certainly regulated by $\varsigma_{{{\text{thres}}}}$ and can be extremely low for example, if $\varsigma_{thres} < < 1$.

New member of the array is generated with random coordinates within the prescribed area $\{ [0,Lx],[0,Ly]\}$ with the mass ($m_{N + 1}$) randomly distributed around a starting (basically small) one $m_{s}$. Qualitatively it means that at every time moment the array can be supplemented with some probability by a new member which is physically placed in some arbitrary place inside the area $\{ [0,Lx],[0,Ly]\}$. At the same time, we have to introduce a process of disappearance of some of the array members. Natural criterion for this will be established below from the checking of the accumulation or loss of the mass (e.g. reserve fat mass) $m_{j}$ by every individual. It is supposed that, if an animal gets critical size (which can be both: maximal $m_{\max }$ or minimal $m_{\min }$) it disappears from the system and the length of the array decreases $N \to N - 1$.

Both of such events must be adjusted numerically to some rate natural for a particular biological system. It is quite expected from the very beginning, that there should be a kind of balance between the creation and disappearance rates. If new animas statistically appear too often, the overpopulation will take place. In opposite limit the array will quickly shrink $N \to 0$ and it will cause a distinction of the population.

Obviously both these rates should be naturally regulated by the available food resources. For the particular problem under consideration the resources are generated by a random deposition of the potentially available food onto the water surface. It means that we introduce a new array for the food with coordinates $\overrightarrow {r}_{n}^{food}$. The length of the array $N^{food}$ is also variable and index $n$ running in the interval $n = 0,..,N^{food}$. In principle, it is possible, and very often happened in our simulations, that at some particular moment available food inside the area can completely disappear. In this case the length of the food array reduces to zero $N^{food} = 0$.

The food is generated by the random deposition of the food portions distributed inside the area of the system $\{ [0,Lx],[0,Ly]\}$. Generally, it is organized in the same manner as the deposition of the new individuals. If the random number $\zeta$ uniformly distributed in the interval [0;1] is less than some threshold $\varsigma_{thres} < 1$ the food portion is deposed at an arbitrary place of the area, in principle at any given time step $\Delta t$.

It is obvious that if the threshold is much smaller than unit: $\zeta_{thres}^{food} < < 1$ the food portions are produced very rarely. Certainly, the rate of the “food production” in the frames of the model must be properly regulated to make its behaviour natural. Of course, it relates to the size of the portions, but also to the intervals between the food depositions. In any case, these intervals are expected to be much longer than discrete time steps $\Delta t$ used to solve numerically the equations of motion. At the same time, it must be much shorter than other (biologically reasonable) time-scales of the problem. To control the stability and reasonability of the simulations we have varied this rate in very wide intervals. It was found that at all the reasonable rates food balance in the system tends to the stationary scenario, which corresponds to the expected scenarios in nature.

When a portion of the food falls onto the surface the animals which occasionally appear relatively close to it are attracted to this food portion and “eat” it with some characteristic rate. In the particular simulation it was simulated by the additional term of the attraction force:

6 $$f_{{_{jn} }}^{food} (\overrightarrow {r}_{j} ,\overrightarrow {r}_{n} ) = B_{jn}^{food} (\overrightarrow {r}_{j} - \overrightarrow {r}_{n} )\exp \left[ { - \left( {\frac{{\overrightarrow {r}_{j} - \overrightarrow {r}_{n} }}{{R_{{_{j} }}^{food} }}} \right)^{2} } \right]$$

As it is seen from the interactions in the model the animals compete for the food, repulsing one another and reacting faster on the force $f_{{_{jk} }}^{food} (\overrightarrow {r}_{j} ,\overrightarrow {r}_{k} )$. It’s why we apply here non-uniform coefficient $B_{{_{jk} }}^{food}$, which is different for different index $j$ and in fact depends on some power of mass $B_{jk}^{food} \sim m_{j}^{\alpha }$ with some exponent $\alpha$. Scaling estimation gives the value of the exponent $\alpha$ = *2/3*. Another form of the competition is related to their simultaneous consumption the food with different rate depending on the size of the different individuals. We suppose that the consumption is proportional to already accumulated mass of the individual: $\partial m_{j} /\partial t = \mu m_{j}$. Effective dumping $\eta_{j}$ on the water surface also depends on the mass of animal $\eta_{j} \sim m_{j}^{\beta }$, where estimated exponent $\beta$ = *1/3*.

Let us remind that the consumption of the food portion with index (*n*) is possible only when the animal is close enough to this portion. In other words, one more threshold has to be incorporated: $\left| {\overrightarrow {r}_{j} - \overrightarrow {r}_{n} } \right| < R_{thres}^{food}$. As we see, the model consists of the dynamic equations of motion and a number of the procedures, which work in parallel and essentially affect both: the dynamic behavior and the results.

The equations of motion can be formally written accumulating all the above mentioned forces of the problem:

7 $$m_{j} \partial^{2} r_{j} /\partial t^{2} + \eta_{j} \partial r_{j} /\partial t = \sum\limits_{k} {\left[ {f_{{_{jk} }}^{repuls} (\overrightarrow {r}_{j} ,\overrightarrow {r}_{k} ) + f_{{_{jk} }}^{attract} (\overrightarrow {r}_{j} ,\overrightarrow {r}_{k} )} \right]} + \sum\limits_{n} {f_{{_{jn} }}^{food} (\overrightarrow {r}_{j} ,\overrightarrow {r}_{n} )} + \sum\limits_{Boundaries} {f_{j}^{bound} }$$

with their solution combined with the procedures described above. All the initial velocities are zero. This combination makes the solution nontrivial because it is performed for the arrays with changeable lengths and with the varied sizes and forces of the participants. Nevertheless, expected scenarios can be generally predicted and verified later by the large set of numerical experiments at different combinations of the parameters.

The longer the time an individual spends near the portion of food and the larger the size it has to the current moment the faster it eats up and faster accumulates additional mass. In general, the larger animals consume more food. However, one should remember that large animals may also have disadvantages. For example, due to inertia of motion faster animals can occasionally jump over a good position near the food. We have observed many such local events during the simulations. Such events partially can be observed in the movies presented below. It is also possible that small animals can appear near the randomly deposed food earlier than the big ones and start consuming it.

In some cases such possibilities even emerges due to not just probabilistic but quite regular reason. For example, it can happen due to a further described effect of the “wave of fear”. By influence of such a wave (being scared by somebody moving along the shore), the animals almost synchronically start to run away from one of the boundaries. Big, strong and fast animals escape far away, while the small ones still remain near to the shore. As result, the food deposed into this region will be partially (or even completely) consumed by the weak individuals before the strong ones will return. This effect will be reported further on.

Basically, dynamic balance of the population is determined by the relation between a set of the time constants of the problem: how often the food appears, how quick the animals consume it and how efficiently they grow. However, the population structure is also important for the total balance. The more an animal consumes the bigger and stronger it becomes, than faster moves to the new portion of the food and more eats. The individuals are born small and more or less equal. They grow initially depending on their “luck” only. However, population quickly forms some non-uniform distribution of the sizes.

In terms of distributions one can say that with the time the individual members of the array shift along the distribution to the larger or smaller size depending on the personal balance of accumulation or losing the mass. At every instant moment the distribution actually demonstrates two opposite fluxes: to the limit of the small and large sizes. Strongest animals evolve to the predefined critical large size $m_{\max }$, at which they leave the population. Weakest ones tend to the critically small weight *m*_min_ and also leave the population. The balance is maintained by the fact that at every time moment the population is incorporated by new young members who either grow or die.

In this respect, it is interesting to observe the dynamics of the animal’s sizes distribution. Obviously, the initial population consisting of young animals has histogram localized around small sizes. Later the distribution becomes wider and its edge moves to the larger sizes. The asymptotic histogram shape is determined by the two opposite processes described above. This shape will be compared further with a real distribution found from the field observations.

Typical behavior of the model evolution for a population inside a limited area is presented in illustrative movie (video_S2). For a convenience, the population is formally divided into 3 subgroups, which are plotted by the circles having different sizes (small, medium, and big) and colors (blue, green, and red, respectively). The randomly deposed portions of food are shown by large black circles.

We start from relatively young population, which contains 100 individuals and allow them to move, grow and disappear according to the rules of game. This behavior demonstrates correlative motions, as well as variation in the population composition, which look self-consistent and rather natural. The interactions between the animals with other animals and with food cause quite predictable motions of the individuals and the changes of their sizes (and colors, respectively) when they leave one of the three groups and join to another. The process stabilizes with the time and seems to become stationary. Below we will study this process by the quantitative time-dependencies and statistical histograms.

The bigger an animal the faster it moves in average. Therefore, it is convenient to sort the animals according to the masses and velocities. Such representation is reproduced in the third movie “video_S3.mp4”. The separation between subgroups, their evolution from the initial to a stationary state is clearly seen from the movie in dynamics. Moreover, one can observe even how group of the small animals divides by itself into two subgroups with quite well pronounced gap between them. This separation is caused by the mentioned above two fluxes of the sub-populations, which either grow from the initially small sizes to the medium ones or “in unlucky case” decrease and disappear.

Actually, the rate of evolution with fast changes of the masses after very few acts of the interaction with deposed food represented in both movies is strongly overestimated in contrast to the reality. Therefore, as a next step we reduced the rates of accumulation and loss of the mass and proportionally prolonged the simulation time. This process was also recorded in two movies “video_S4.mp4” and “video_S5.mp4”. To reduce the lengths of the videos the time intervals between the frames were made 10 times longer. Because of this reduction the movies look almost stroboscopic. Nevertheless, the movies illustrate quite well long-time dynamics of the system at realistic rate of food deposition and consumption.

**Figure 1:**
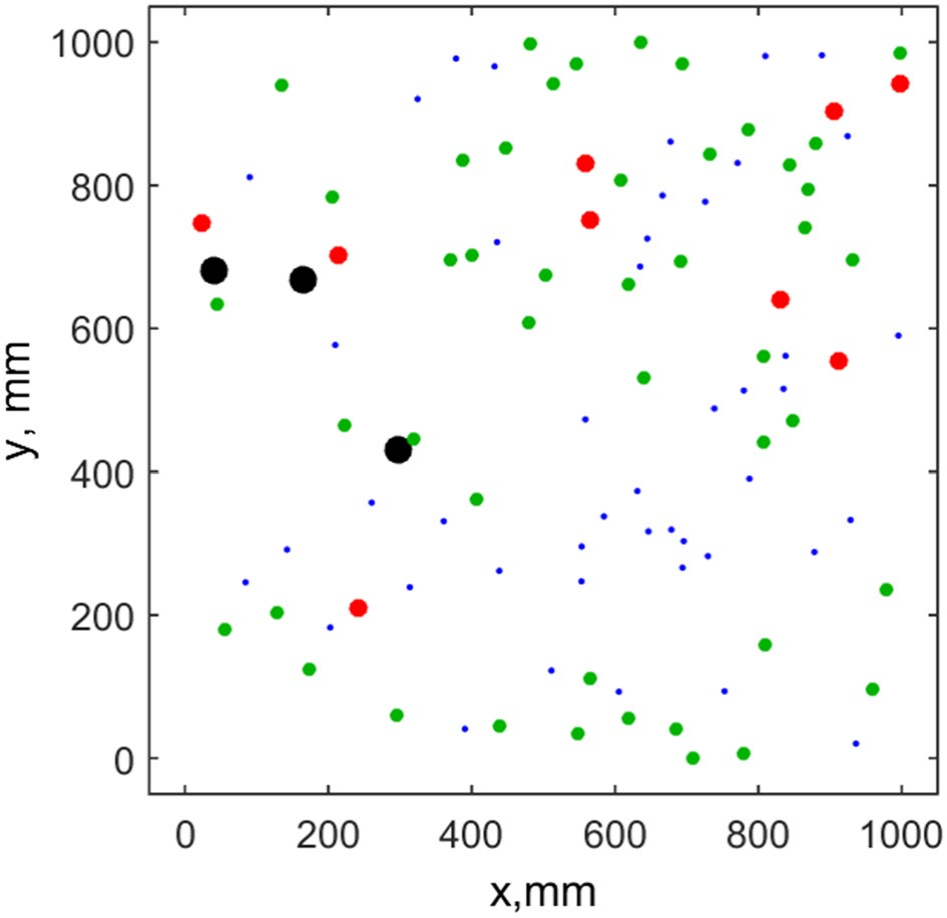
Typical spatial pattern formed by the population at intermediate stage of evolution. Blue, green and red circles correspond to the small medium and large sizes of the individuals. One can see the groups packed by the individuals with the distances close to the equilibrium *R*^min^$R^{\min }$ and voids. Black circles show food portions remaining to the current moment.

**Figure 2:**
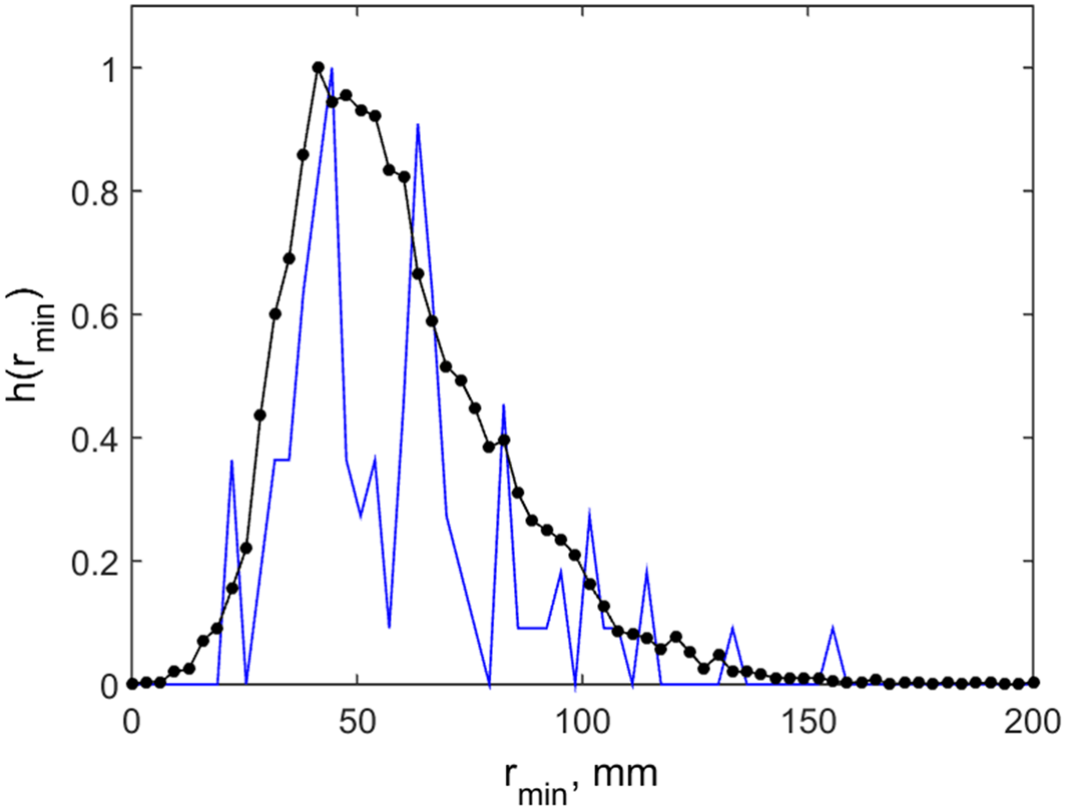
Histograms of the distances between nearest neighbors. Blue line shows some instant distribution. Black line presents histogram accumulated during long run. Maximum of the histogram corresponds to the distance *R*^min^ close to the equilibrium. Long tail of the black curve corresponds to the presence of some individuals inside the voids.

**Figure 3:**
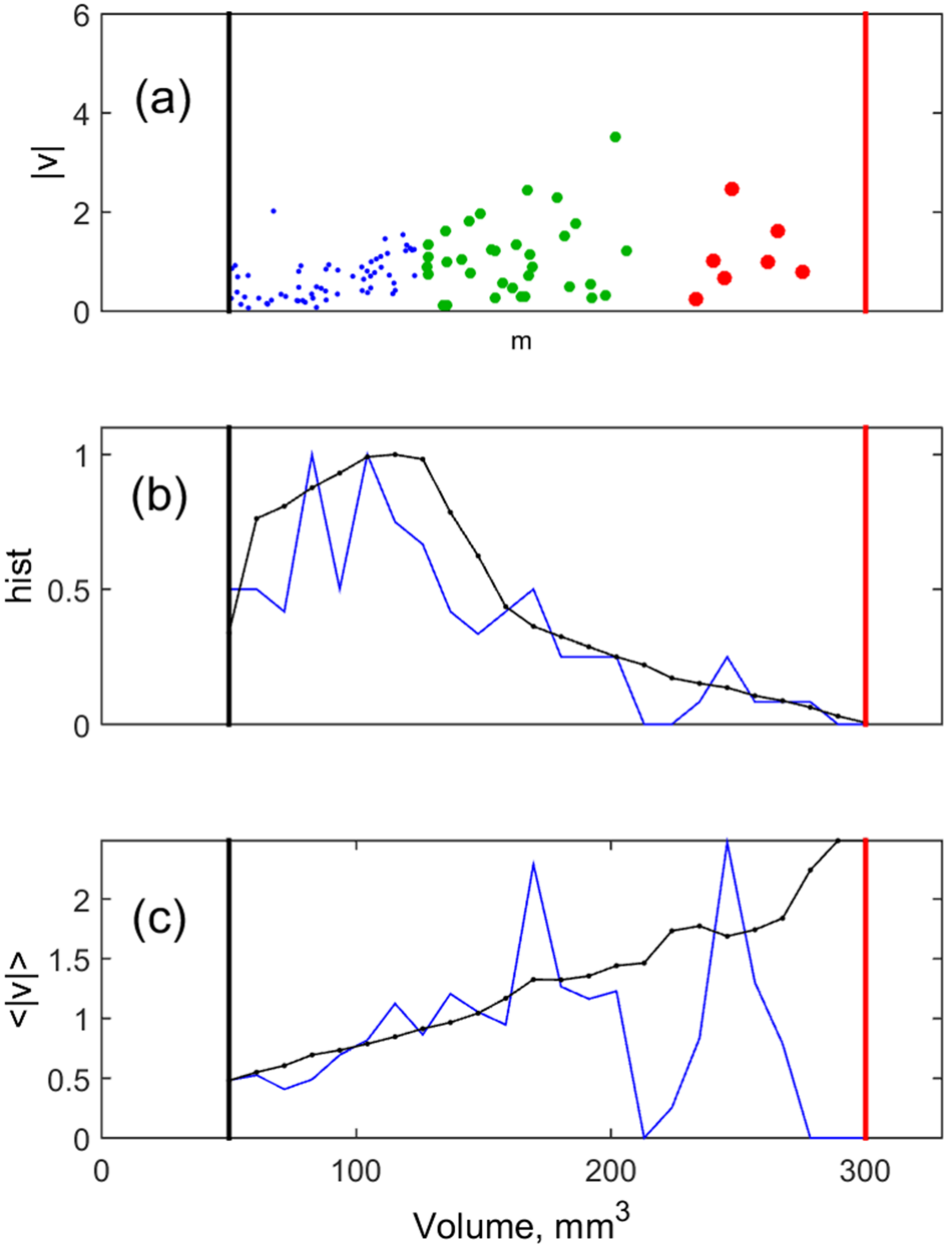
Mass depending values: (**a**) instant configuration of the velocities of different subgroups marked by the same symbols as in Fig. 1; (**b**) instant and long run averaged histograms of the masses for complete population; (**c**) instant and accumulated distributions of the velocities monotonously increasing with masses (volumes) of the individuals.

**Figure 4:**
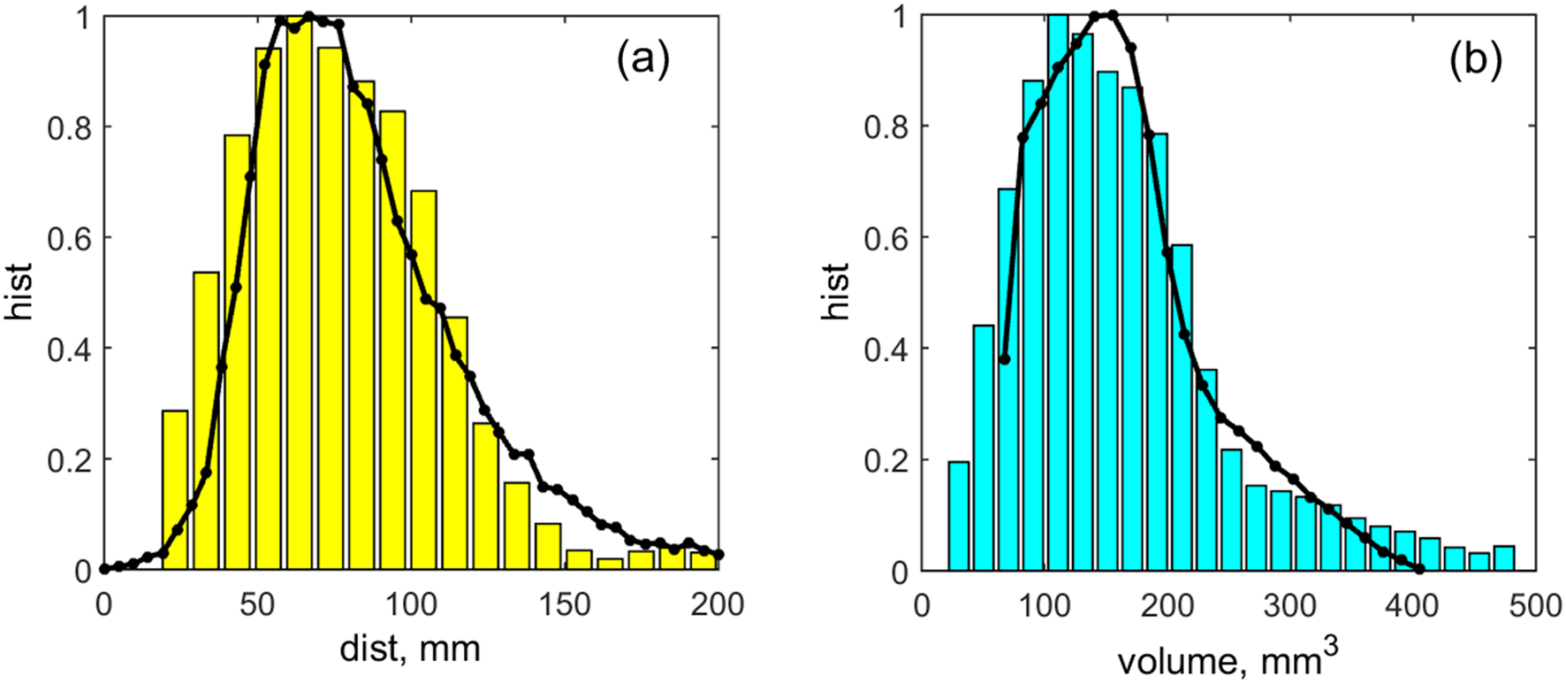
The comparison between the numerically (curves with points) and experimentally (bars) obtained data. The subplot (**a**) shows the histogram of the distances between nearest neighbors and the subplot (**b**) presents the histograms of the volumes of the animals (which are supposed to be approximately proportional to their masses).

Information about the system accumulated during long runs including typical pattern formed by the moving animals at some intermediate stage and other plots is presented in Figs. 1, 2, 3 and 4. The spatial pattern in Fig. 1 is taken at some intermediate stage of evolution. Blue, green and red circles correspond to the small medium and large sizes of the individuals. Large black circles mark the food portions, which are recently deposed and not eaten yet.

One can well distinguish in Fig. 1 relatively dense domains packed with the distances between the individuals close to the equilibrium radius $R^{\min }$ determined by the relation between repulsive $f_{{_{jk} }}^{repuls} (\overrightarrow {r}_{j} ,\overrightarrow {r}_{k} )$ and attraction $f_{{_{jk} }}^{attract} (\overrightarrow {r}_{j} ,\overrightarrow {r}_{k} )$ forces. These domains are separated by voids. To characterize such patterns quantitatively the histograms of the distances between nearest neighbors^6^ were accumulated during a long simulation run. These histograms are shown in Fig. 2. Thin blue line here reproduces some arbitrary instant distribution. Black line with the circles presents the histogram accumulated during a long run. Maximum of the histogram is close to the equilibrium distance $R^{\min }$ determined by the balance of interactions. Long tail of the black curve, which extends to the few times longer distances than $R^{\min }$, exists, since some individuals are located inside voids far-away from all the neighbors.

Typical size distributions to which the population evolves with the time are reproduced in Fig. 3. It shows three important size depending values. Figure 3a demonstrates an instant configuration of the velocities. Three different subgroups are marked by the same symbols as in Fig. 1. Figure 3b shows instant histogram of the animal’s masses in complete population and the histogram averaged over long run. They are plotted by the blue and black lines respectively. How mean velocities of the animals depend on their mass is shown in Fig. 3c. As above, the instant and accumulated distributions are plotted by the blue and black lines respectively.

It can be noticed from the subplot (c), Fig. 3, that the averaged velocities correlate with the mass of the individuals. In principle, it could be expected according to the model rules, namely due to the nonlinear dependence of strength $f_{{_{jk} }}^{repuls} (\overrightarrow {r}_{j} ,\overrightarrow {r}_{k} )$ as well as effective damping $\eta_{j}$ for the individual animals on their size (mass $m_{j}$). However, taken into account multiple interactions in the system the result was not obvious in advance. The velocity increase is especially pronounced when the animals become large and are close to the final stage of its growth.

Further, we would like to compare the numerical and experimentally obtained data. This comparison is presented in Fig. 4 by the curves and bar-plots respectively. From the field videos it is difficult to determine the masses $m_{j}$ of the individuals, but we can estimate their volumes $V_{j}$ which are approximately proportional to their masses $V_{j} \sim m_{j}$. Therefore, to compare numerical results with the measurements from videos we have plotted the distributions along the volume coordinate. The subplot (a) in Fig. 4 shows the histograms of the distances between nearest neighbors, and the subplot (b) presents the histograms of the volumes of the animals. The histograms obtained from simulations match very well to the histograms calculated using experimental data. Two sample Kolmogorov–Smirnov test does not reject the hypothesis, that the distributions obtained in experiment and in numerical simulations are from the same continues distribution for both the animal volumes (*p* = 0.59, D_55,500_ = 0.105) and for the distances between nearest neighbors (*p* = 0.84, D_55,500_ = 0.083).

**Figure 5:**
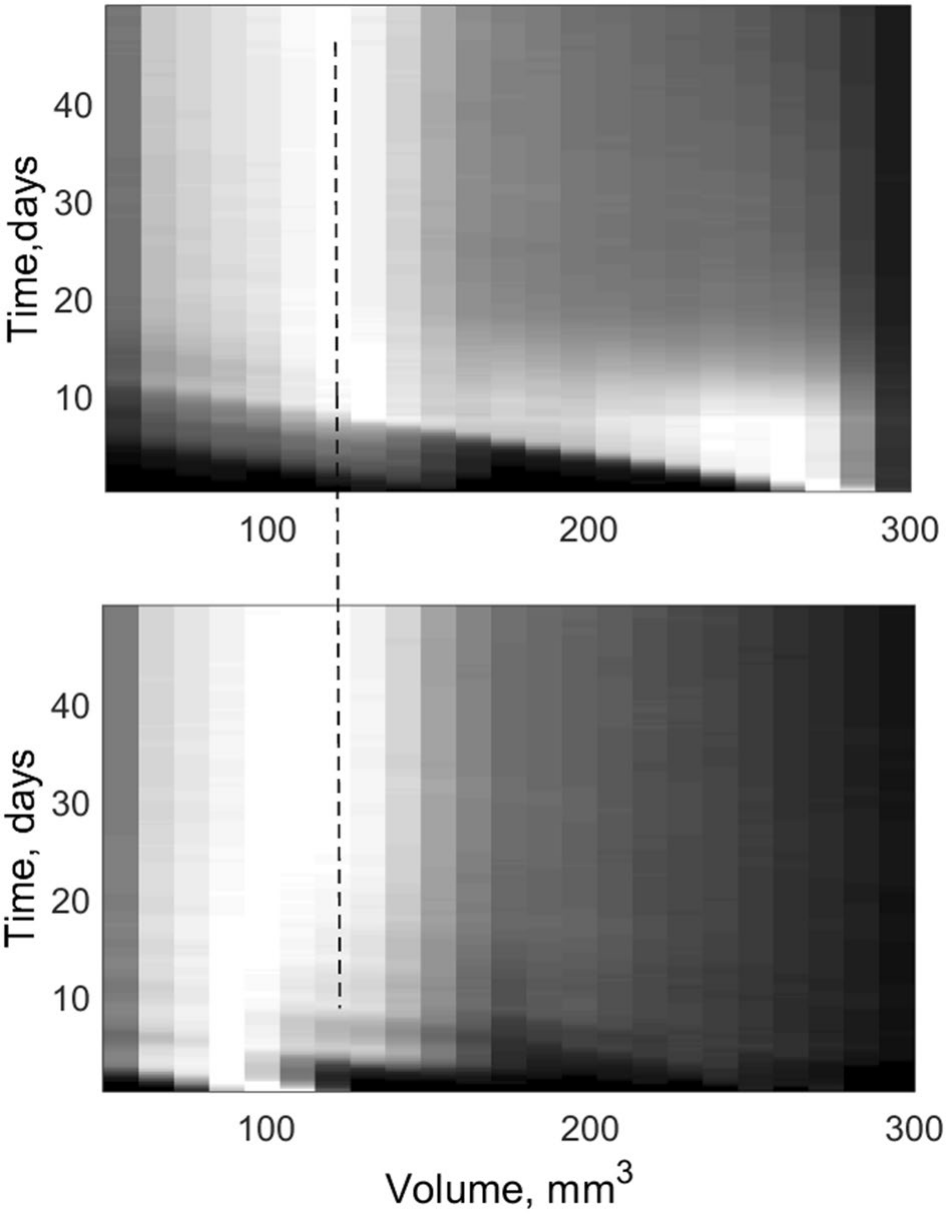
Gray-scale maps of time-volume histograms accumulated for two limit cases: starting from large and small animals, are shown in the subplots (**a**,**b**) respectively. Lighter gray color corresponds to the higher density. Dashed line marks common position of distribution maximums to which they converge during extremely long runs.

**Figure 6:**
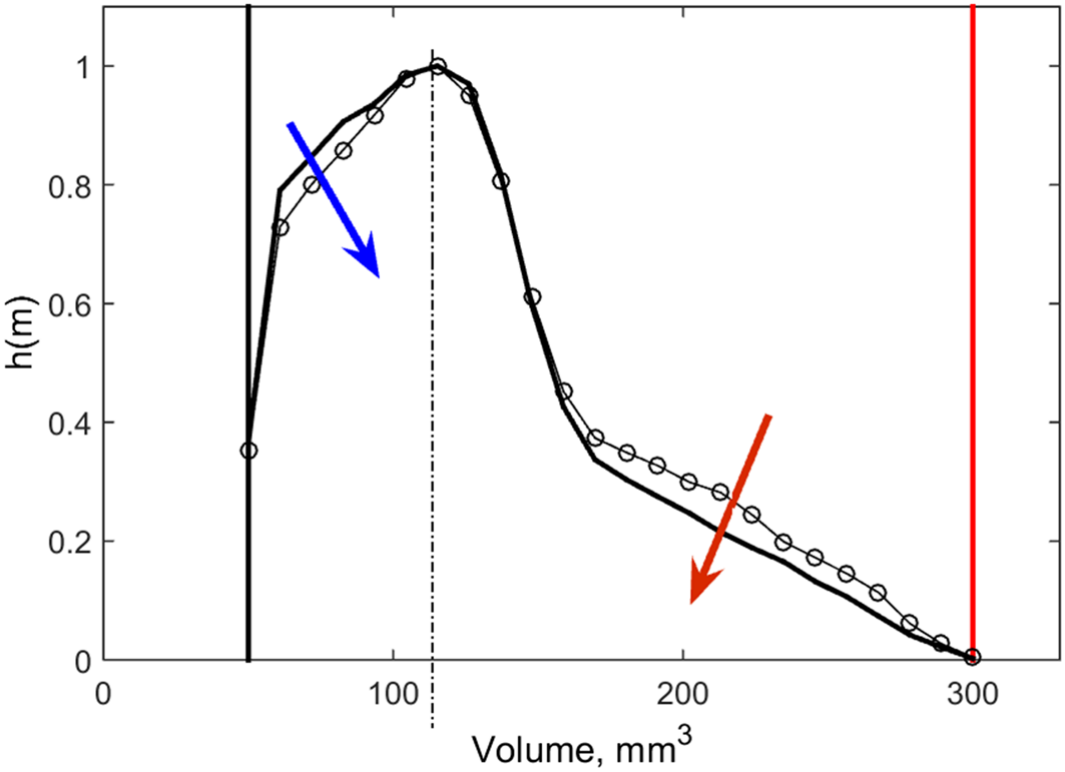
Final volume distributions after long runs. It is seen that the maximums coincide already. Remaining trends of the histogram alterations for the distribution started from the large (open circles) and small (closed circles) individuals are marked by the red and blue arrows respectively.

It is important to note that due to stability of the system the distributions independent on their initial shape converge to the same quasi-stationary histograms. To check this convergence, we started from two extreme populations. The first one consisted almost from the large individuals only and another one contained only small individuals. Time depending volume histograms were accumulated into the volume distribution over time gray-scale maps. In Fig. 5 the results for the two cases are shown in the subplots (a) and (b) respectively. Lighter gray color corresponds to the higher density. Both distributions attract to the practically the same final one with the time. The shapes of distributions, which have started from the two extreme initial distributions, almost coincide after long runs, while the distributions flanks shift in different directions as marked by the red and blue arrows in Fig. 6 and visualized in the supplementary movies “video_S6.mp4” and “video_S7.mp4” respectively. As in the static figures the instant and time averaged histograms are shown by the blue and black lines respectively. For the supplementary video “video_S6.mp4” the distribution is initially localized mainly near the right side of the interval and after some quick transient period “jumps” to the distribution close to the final histogram. Comparison with second video “video_S7.mp4” shows how both averaged distributions are attracted after a long simulation run to the similar distribution.

**Figure 7:**
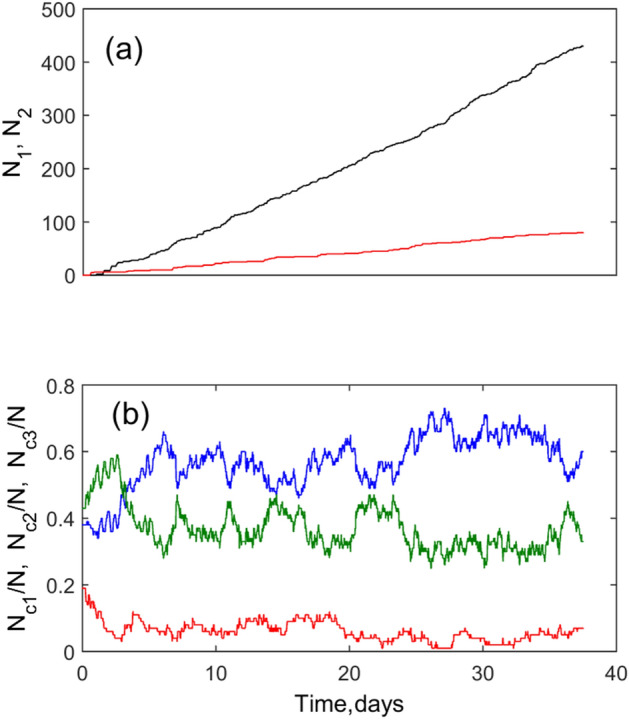
Time depending integral populations: (**a**) accumulated numbers of died (*N*_1_) and survived (*N*_2_) animals (black and red curves); (**b**) variations of the numbers of animals in subpopulations of small (*N*_c1_), medium (*N*_c2_) and large (*N*_c3_) animals shown by blue, green and red curves respectively.

During the system evolution some of the animals leave the population and some new appear. One can accumulate the numbers of died and survived animals for each given time moment. Accumulated numbers of died and survived animals are plotted in Fig. 7a by black and red curves respectively. The balance between these numbers depends on the relation of all the model parameters. Here the same set of the parameters was used as for simulations presented in Figs. 1, 2 and 3.

The curves presenting the integral number of animals are relatively smooth, Fig. 7a, yet the instant numbers of animals in small, medium and large subpopulations shown by blue, green and red curves respectively in Fig. 7b vary much stronger, because at every particular time moment, their numbers are relatively small and the fluctuations are strong. From the figure, it is also obvious that during initial transient time interval the number of small individuals do not exceeds the number of two other populations. It is natural and was expected, because all newborn animals are small. Therefore, subpopulation with small size prevails at stationary stage.

As we already announced nontrivial transformation of the spatial pattern and modification of all the resulting distributions can appear due to the “waves of fear”. Such a wave can appear when the animals almost synchronously try to escape from the shoreline if they are being scared by somebody moving along. Mathematically such a wave can be provoked by an additional repulsion force acting from one side of the simulation area to the distance much longer than already incorporated short range reflection from the boundary (Eq. 6). This force influences all the members of the array but appears for relatively limited time. It can be added to the model interaction occurring either randomly or periodically. Second variant is more regular and preferable and allows accumulate statistics faster.

Analytically this force has the same form as $f_{j}^{bound}$ with the same boundary position $\overrightarrow {r}_{bound}$:

8 $$f_{j}^{wave} = B_{j}^{wave} \exp \left[ { - \left| {\frac{{\overrightarrow {r}_{j} - \overrightarrow {r}_{bound} }}{{R_{{}}^{wave} }}} \right|} \right],$$

but it has larger amplitude $B_{j}^{wave} > B_{j}^{bound}$ and much longer distance of the exponential decay $R_{{}}^{wave} > > R_{{}}^{bound}$. So, it influences not only the individuals occasionally attempting to cross the boundary but almost all others too, even if they are currently relatively far from the boundary.

The video in supplementary material “video_S8.mp4” illustrates typical behavior of the system at presence of the “waves of fear” occasionally observed in original sequences (video_S1.avi). One can see how bigger, stronger and faster animals escape quicker and further from the dangerous boundary, while some smallest ones do not react so quickly and remain almost near to it. Food deposition is not correlated with the “waves of fear”. As result, the food deposed near the dangerous boundary is consumed mostly by small animals, since that boundary region is practically depopulated by bigger animals. While stronger individuals return to the shore, such food may be already consumed by the weaker individuals.

**Figure 8:**
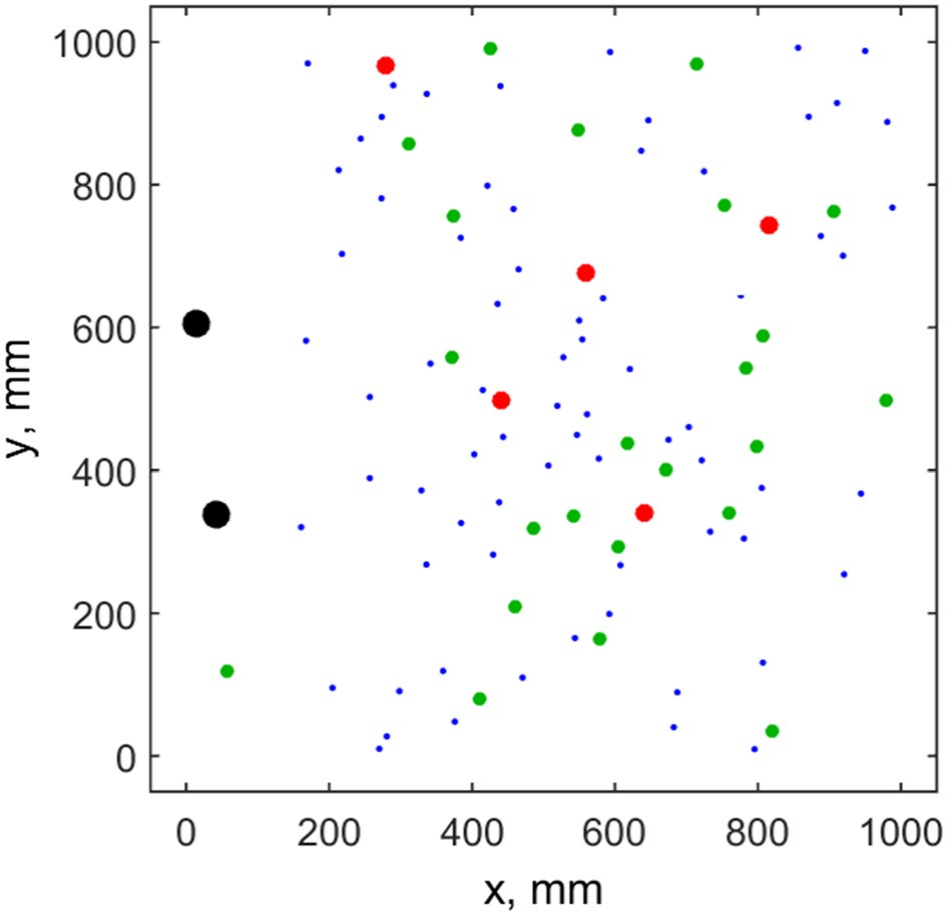
Shifted pattern at the presence of the “wave of fear”. Some food was just deposed inside the depopulated area near to the dangerous boundary. Some small individuals are in the vicinity to the food, while absolute majority of the population is localized in a center or near opposite boundary. The symbols have the same meaning as in Fig. 1.

Static image of such “shifted” to one side pattern for a moment when food and weak members of the array are presented in one side of the area while absolute majority of the population is collected in a center or closer to another side of the area is reproduced in Fig. 8.

**Figure 9:**
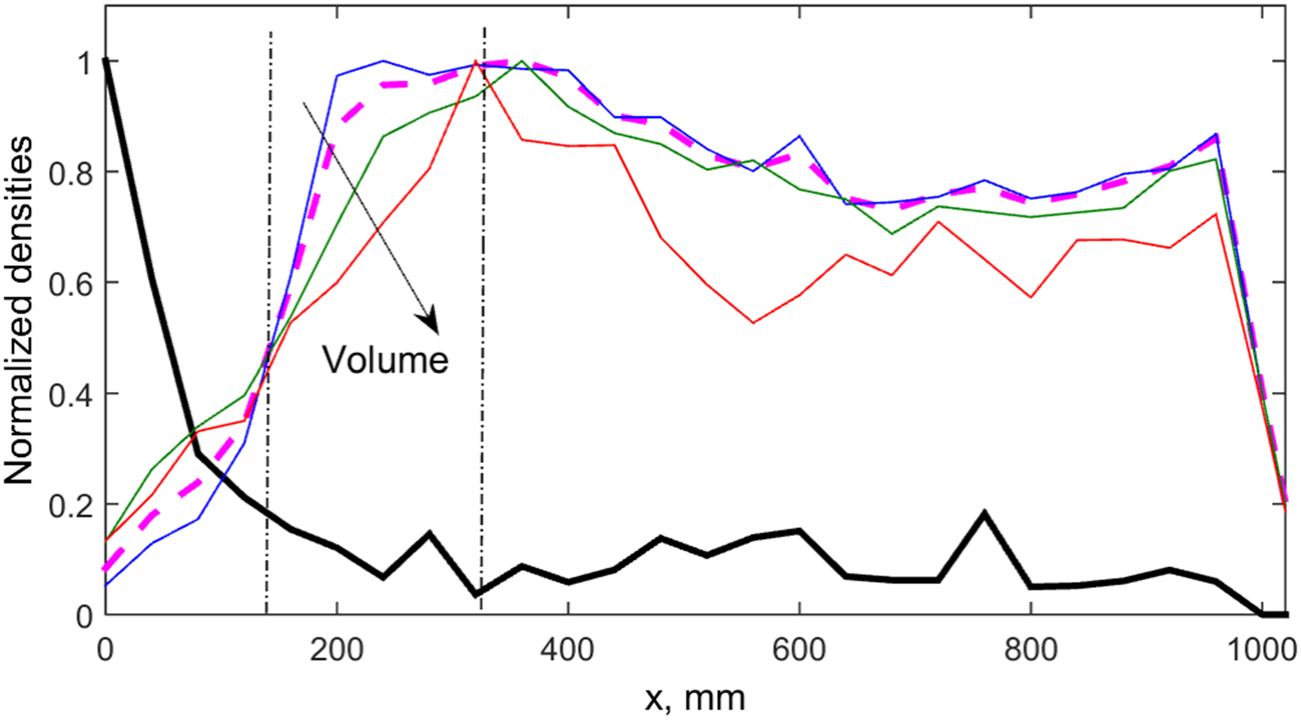
Density distributions of the food and animals at presence of regularly applied “waves of fear”. The densities of the food and animals are shown by the solid black and dashed magenta curves respectively. The curves for the densities of large, middle and small individuals are plotted by the same colors as above. The density shift with increasing size is marked by the arrow.

Being regularly applied the “waves of fear” in principle leads to a redistribution of the food and animals in the space. It is quite expected that the density of the animals will spend some time in the areas distanced from the dangerous wall. As result, the food will longer remain available in the places close to the dangerous wall. We have accumulated these densities during long runs at different parameters and obtained such shifted distributions. Typical distributions of the food and animals for the set of parameters which were used in simulations presented in the previous figures are shown in Fig. 9.

Mean density of the food is shown by the solid black curve. Total density of the animals is presented by the dashed magenta curve. These densities are anti-correlated. Besides, there is a fine structure of the partial densities of the animals. The curves for the densities of large, middle and small individuals are plotted by the same colors as above. Strongest correlation between the size of the animals and their spatial density is observed in interval between two dash-dotted lines. The density shift with increasing size is marked by the arrow. In close proximity to the dangerous wall, where mean density of the food is abnormally high, the density shift dependence on the individuals size change the direction. Number of the small individuals reduces in average, because they quickly move to the category of middle or even large individuals.

## Conclusions

The presented long-time mechanistic swarm model of water striders demonstrates the realistic population dynamics. For the swarm formation in the model, attraction and repulsion forces are responsible. Animal motion includes inertia and kinetic energy dissipation effects. The model includes three different rates related to the growth of individuals: appearance rate of the discrete food, food assimilation (animal growth) rate, stored energy (mass in the model) loss rate. Simulated population has fixed appearance rate of young animals. Young weak animals (individuals having stored energy below some critical value) disappear from the population. They can die because of the hunger or can be eaten by larger individuals^7^. Adult animals can also leave the simulated population.

Basically, young animals experience the highest death risk, because of the strongest competition for the resources, especially with large individuals. Small animals with low mass are forced out by animals with large mass from food sources. Besides, our observations and simulations demonstrated the presence of correlation between animal size and their motion velocities. Therefore, animals with larger mass reach food quicker. However, in some situations, small animals may profit from their low motion velocity. If, for example, a potentially dangerous event takes place at some area, the small animals escape that area slower and may potentially gain trophic advantages in that area. This phenomenon was clearly observed in the model. Also, “the waves of fear” observed in the nature, when animals escape from dangerous place and re-occupy it after a while, could be well observed in the model.

The histograms of individual sizes and distances between nearest neighbors were demonstrated to be an excellent tool for characterization of long-term system dynamics. Size distribution seems to be an adequate measure for population status, and it has a characteristic shape for different model parameter combinations. Distribution of the distances between nearest neighbors is other important measure of the population density and its dynamics. Parameters of the model can be tuned in such a way, that the shape of both distributions in a steady phase coincides with that shape observed in a natural population, which help us to understand the factors leading to particular momentary distribution of both parameters (size and distance) in the population. From this point of view, the model can provide some predictions: how both distributions can further develop from certain state depending on particular combination of factors.

Besides the statistical information accumulated during long time runs of the model at different relations between all the parameters one gets an option to observe a fine structure of the dynamic processes and compare it with the intuitively expected structure. It allows study various scenarios of the behavior and test different hypotheses about the system structure and evolution even at a lack of the experimental information about it. Clear visual control allows directly observe from the one side the correlations between the structure of population, its motion and patterns formed in physical space, collisions between the animals competing for the food; and from another hand it allows to observe evolution of the calculated histograms, total population, etc.

## Supplementary Information

Supplementary Figure S1.

Supplementary Figure S2.

Supplementary Legends.

Supplementary Video 1.

Supplementary Video 2.

Supplementary Video 3.

Supplementary Video 4.

Supplementary Video 5.

Supplementary Video 6.

Supplementary Video 7.

Supplementary Video 8.
