# Supplementary figures and images for: Numerical model of the spatio-temporal dynamics in a water strider group
Source: Sci Rep. 2021 Sep 10;11:18047. doi: 10.1038/s41598-021-96686-w (PMC8433171; doi:10.1038/s41598-021-96686-w)

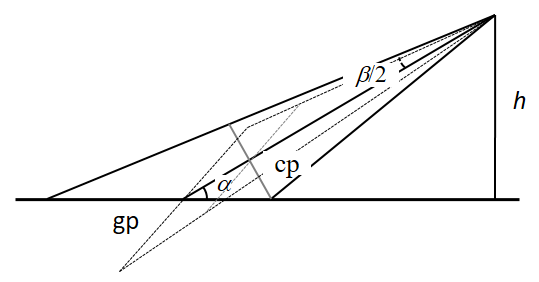

Supplement: Supplementary file 1 — Supplementary Figure S1. [file 41598_2021_96686_MOESM1_ESM.png]

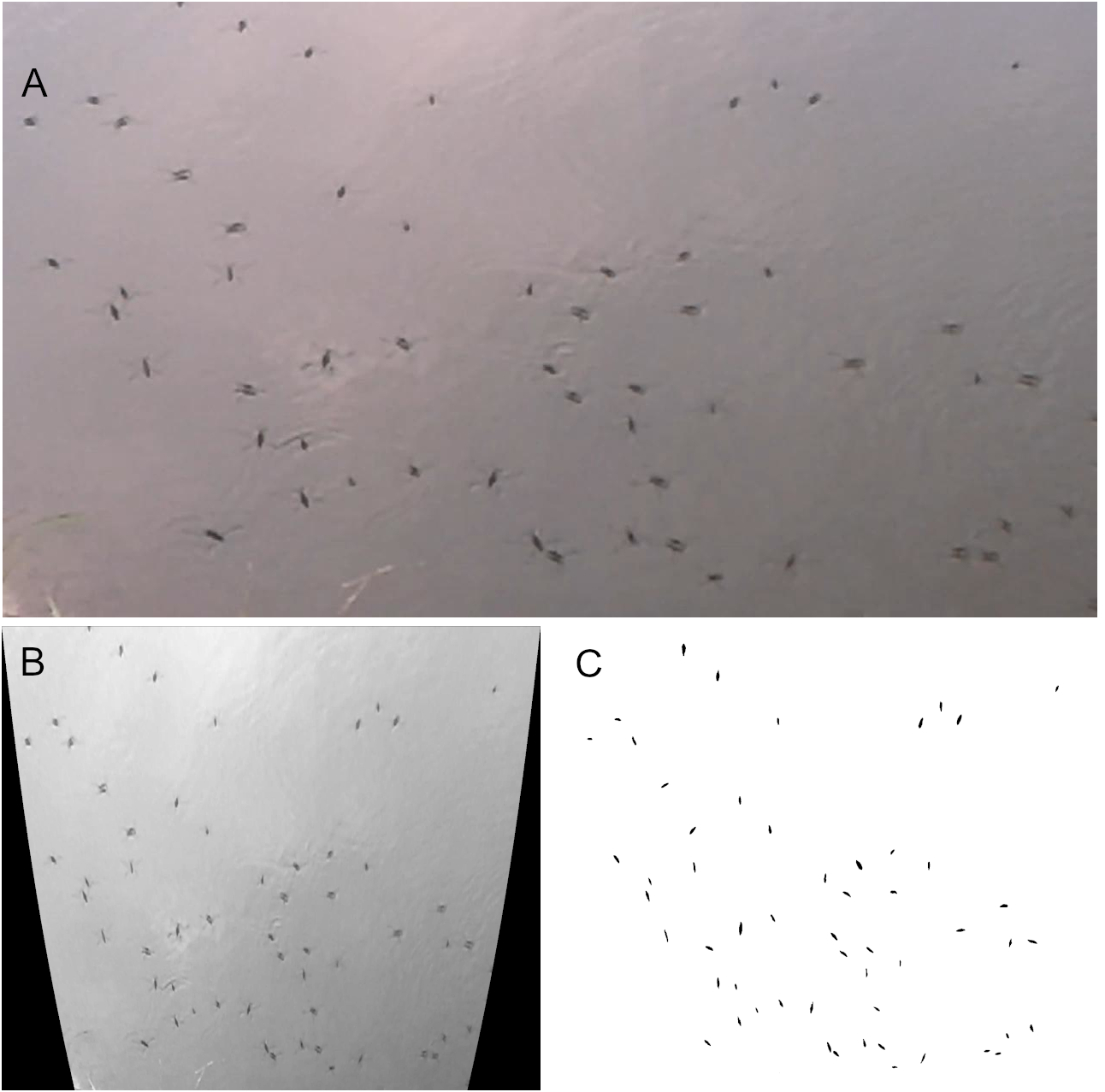

Supplement: Supplementary file 2 — Supplementary Figure S2. [file 41598_2021_96686_MOESM2_ESM.png]
