# Supplementary material for: Numerical model of the spatio-temporal dynamics in a water strider group
Source: Sci Rep. 2021 Sep 10;11:18047. doi: 10.1038/s41598-021-96686-w (PMC8433171; doi:10.1038/s41598-021-96686-w)
Supplement: Supplementary file 3 — Supplementary Legends. [file 41598_2021_96686_MOESM3_ESM.docx]

**Numerical model of the spatio-temporal dynamics in a water strider group**

Alexander Kovalev, Alexander E. Filippov, and Stanislav N. Gorb

**Supplementary Information**

**Supplementary figures**


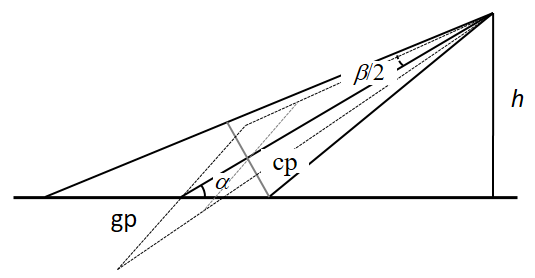


**Fig. S1.** Projection of the camera plane (cp, shown in gray) to the ground plane (gp). The angle between the normal to the camera plane and the ground plane is *α*, the half angle of view in horizontal direction is *β*/2, the height of the camera above ground plane is *h*. The direction perpendicular to the page is shown by dashed line.


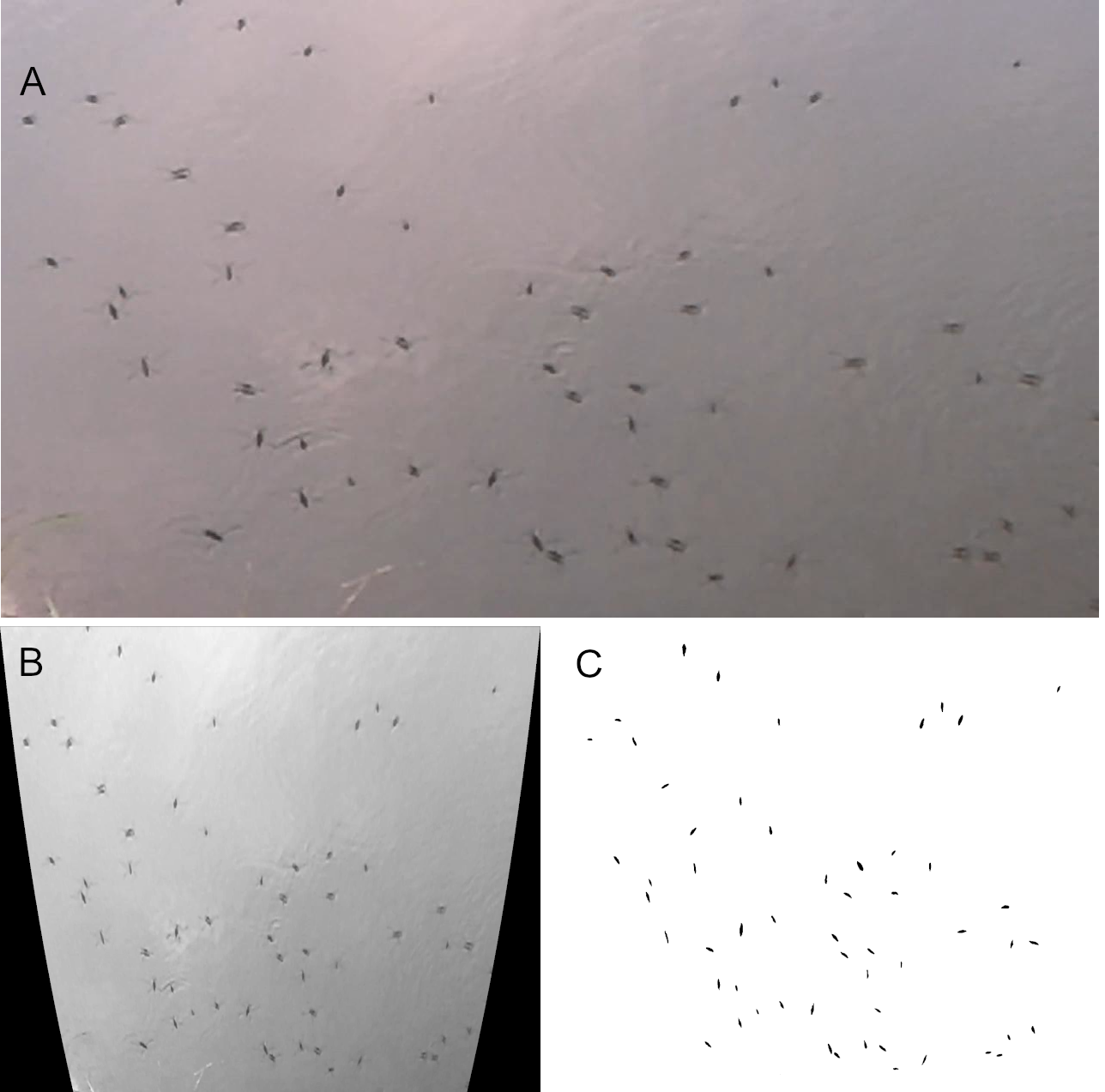


**Fig. S2**. Processing of a camera image (A). (B) the water surface in real coordinates after fitting of the observation angle (the angle between the normal to the camera plane and the ground plane). (C) binary image of water striders painted over using GIMP.

**Supplementary movies**

**video_S1.avi**

Original video recording of the typical behavior of the water strider group. This kind of video documentation was analyzed and used for establishing numerical model presented in this paper. In the first part of the sequence, an undisturbed behavior of the animals can be observed. It is well seen that behavior of larger and smaller individuals is rather distinctive. In the second part of the sequence, the behavior under influence of potential predator can be observed. The collective behavior of numerous individuals in the group leads to the appearance of so-called “waves of fear” that are modeled and explained later in our model.

**video_S2.mp4**

Typical system evolution reproduced in dynamics of the population inside limited area on the water surface. For a convenience, the population is formally divided into three subgroups which are plotted by the circles having different sizes (small, medium and big) and colors (blue, green and red), respectively. The randomly deposed portions of food are shown by the large black circles.

**video_S3.mp4**

The same process as in the movie “video_S2.mp4” presented in the form of the objects sorted according to the masses and velocities. The separation between subgroups and their evolution from the initial population to a stationary one is clearly seen in dynamics. One can also observe how the group of the small animals divides by itself into two subgroups with quite well pronounced gap between them. This separation is caused by the two fluxes of the sub-populations where animals either grow from the initially small sizes to the medium ones or decrease.

**video_S4.mp4**

The same process as presented in the movie “video_S2.mp4”, but recorded for 10 times lower rates of accumulation and loss of the mass. The simulation time is 10 times longer, the time interval between the frames was 10 longer than in the movie “video_S2.mp4”. Because of this the movie looks as almost stroboscopic one. However, it illustrates well long-time dynamics of the system at realistic rate of food deposition and consumption.

**video_S5.mp4**

The same process as in the movie “video_S4.mp4” presented in the form of the objects sorted according to the masses and velocities.

**video_S6.mp4.**

Dynamic convergence of the distribution starting from the population of large animals to the final, almost static, histogram. Instant distributions are shown by the blue thin lines. Time averaged distributions are plotted by the black curves with the dots. It is seen that initially the distribution is mainly localized near the right side of the interval, but after some quick transient process “jumps” to the distribution close to the final histogram.

**video_S7.mp4.**

Dynamic convergence of the distribution starting from the population of large animals to the final, almost static, histogram. The colors of the curves are the same as in the previous movie “video_S6.mp4”. Direct comparison between these movies shows how both averaged distributions are attracted to the very similar curves.

**video_S8.mp4**

Typical behavior of the system at presence of the “waves of fear”. One can see how bigger, stronger and faster animals escape quicker and further from the “dangerous wall” while some small ones do not react so quickly and remain almost near the dangerous shore. It is also seen that the deposition of the food is not correlated with the periodic “waves of fear”. The food still continues to be deposed near to the “dangerous wall” during the periods when this region is practically depopulated. During such periods the food here may be preferably consumed by the weaker individuals.
